# Comparable Instrumented Knee Joint Laxity and Patient-Reported Outcomes After ACL Repair With Dynamic Intraligamentary Stabilization or ACL Reconstruction: 5-Year Results of a Randomized Controlled Trial

**DOI:** 10.1177/03635465221117777

**Published:** 2022-08-25

**Authors:** Johannes Glasbrenner, Michael J. Raschke, Christoph Kittl, Elmar Herbst, Christian Peez, Thorben Briese, Philipp A. Michel, Mirco Herbort, Clemens Kösters, Benedikt Schliemann

**Affiliations:** † Department of Trauma, Hand and Reconstructive Surgery, University Hospital Münster, Münster, Germany; ‡ OCM Munich, Munich, Germany; § Department of Traumatology and Orthopedics, Maria-Josef-Hospital Greven, Greven, Germany; Investigation performed at the Department of Trauma, Hand and Reconstructive Surgery, University Hospital Muönster, Muönster, Germany

**Keywords:** anterior cruciate ligament, ACL repair, dynamic intraligamentary stabilization, ACL reconstruction

## Abstract

**Background:**

Technical innovation has led to the renaissance of anterior cruciate ligament (ACL) repair in the past decade.

**Purpose/Hypothesis:**

The present study aimed to compare instrumented knee joint laxity and patient-reported outcomes (PROs) after ACL repair with those after primary ACL reconstruction for acute isolated ACL tears. It was hypothesized that ACL repair would lead to comparable knee joint stability and PROs at 5 years postoperatively in comparison with ACL reconstruction.

**Study Design:**

Randomized controlled trial; Level of evidence, 1.

**Methods:**

A total of 85 patients with acute ACL tears were randomized to undergo either ACL repair using dynamic intraligamentary stabilization (DIS) or primary ACL reconstruction with a semitendinosus tendon autograft. The primary outcome was the side-to-side difference in anterior tibial translation (ΔATT) assessed by Rolimeter testing at 5 years postoperatively. Follow-up examinations were performed at 1, 2, and 5 years. PROs were assessed using the Tegner activity scale, the International Knee Documentation Committee (IKDC) subjective score, and the Lysholm score. Furthermore, the rates of recurrent instability, other complications, and revision surgery were recorded. A power analysis was performed a priori, and the Friedman test, Mann-Whitney *U* test, and Bonferroni correction were applied for statistical comparisons with significance set at *P* < .05.

**Results:**

The mean age at inclusion was 28.3 ± 11.5 years in the ACL repair group and 27.1 ± 11.5 years in the ACL reconstruction group. At 5 years postoperatively, a total of 64 patients (ACL repair: n = 34 of 43 [79%]; ACL reconstruction: n = 30 of 42 [71%]) were available for follow-up. At 5 years, ΔATT was 1.7 ± 1.6 mm in the ACL repair group and 1.4 ± 1.3 mm in the ACL reconstruction group (*P* = .334). Preinjury PROs were restored as soon as 1 year after surgery and plateaued until 2 and 5 years postoperatively in both groups. At the 5-year follow-up, the mean Lysholm score was 97.0 ± 5.4 versus 94.5 ± 5.5 (*P* = .322), respectively, and the mean IKDC subjective score was 94.1 ± 9.9 versus 89.9 ± 7.8 (*P* = .047), respectively, in the ACL repair group versus ACL reconstruction group. At 5 years postoperatively, 12 patients in the ACL repair group (35%; age <25 years: n = 10/12; Tegner score ≥7: n = 10/12) had recurrent instability, of whom 10 underwent single-stage revision ACL reconstruction. In the ACL reconstruction group, there were 6 patients with recurrent instability (20%; age <25 years: n = 6/6; Tegner score ≥7: n = 5/6); however, in 5 patients, staged revision was required. Differences between both groups regarding recurrent instability (*P* = .09) or ACL revision surgery (*P* = .118) were not statistically significant. Recurrent instability was associated with age <25 years and Tegner score >7 in both groups.

**Conclusion:**

At 5 years after ACL repair with DIS, instrumented knee joint laxity and PROs were comparable with those after ACL reconstruction. Although no significant difference was found between repair and reconstruction, a critical appraisal of the rates of recurrent instability (35% vs 20%, respectively) and revision surgery (38% vs 27%, respectively) is needed. Young age and a high preinjury activity level were the main risk factors for recurrent instability in both groups. However, single-stage revision ACL reconstruction was possible in each case in the ACL repair group. Although ACL reconstruction remains the gold standard in the treatment of ACL tears, the present study supports the use of ACL repair with DIS as a feasible option to treat acute ACL tears in patients aged ≥25 years with low to moderate activity levels (Tegner score <7).

**Registration:**

DRKS00015466 (German Clinical Trials Register).

Since the early history of the surgical treatment of anterior cruciate ligament (ACL) tears, many efforts have been made to restore knee joint kinematics with ACL repair.^14,20,32^ However, in 1976, Feagin and Curl^14^ reported unsatisfying results at 5 years after ACL repair in 64 cadets at the United States Military Academy: more than 90% of 32 re-evaluated patients reported persistent subjective instability. Improved results after ligament augmentation^8,10,25^ led to a paradigm change toward ACL reconstruction, which is considered the gold standard treatment for ACL tears.^10,37^ Nevertheless, innovative operative techniques with favorable short-term results have led to the renaissance of ACL repair in the past decade.^20^

Although early ACL repair techniques consisted of arthrotomy with open suturing of the ACL with femoral drill holes and cast immobilization for 4 to 6 weeks,^14,32^ modern techniques of arthroscopic ACL repair include refixation using suture anchors,^7^ biological augmentation of ACL repair,^36^ rigid augmentation with suture tapes,^21^ and dynamic intraligamentary stabilization (DIS).^9^ With DIS, the torn ACL is reattached to its femoral insertion, and a nonresorbable cordlike suture is placed along the ACL and fixed to a dynamic spring in an implant in the proximal tibia. Dynamic stabilization of the tibia in a posterior drawer position through full range of motion aims to protect the reattached ACL during the period of healing.^9^

The outcomes of suture anchor repair or suture tape augmentation have been reported only in a limited number of noncomparative cohort studies.^7,21,24,46^ In contrast, favorable patient-reported outcomes (PROs) with sufficient healing of the ACL after DIS have been found in numerous cohort studies, which are summarized in a recent review regarding ACL repair with DIS.^1^ There have been 2 recently published randomized controlled trials (RCTs) that reported on favorable anterior tibial translation (ATT) and PROs after ACL repair with DIS in comparison with ACL reconstruction in the short term.^22,28^ However, there is still a lack of comparative midterm and long-term studies, and the level of evidence to support the use of new ACL repair techniques is still low.^1,20,37^

The aim of the present study was to assess instrumented knee joint laxity and PROs at 5 years after ACL repair in comparison with primary ACL reconstruction for acute isolated ACL tears. It was hypothesized that ACL repair with DIS would lead to instrumented knee joint laxity and PROs that are comparable with ACL reconstruction, with comparable rates of recurrent instability and revision surgery.

## Methods

A single-center RCT was performed. Institutional review board approval was obtained before the study (2013-414-f-S) and registered on the World Health Organization’s International Clinical Trials Registry Platform (DRKS0 0015466). In 2014 and 2015, patients aged between 18 and 50 years with the clinical diagnosis of an acute injury of the ACL (recent trauma with positive Lachman or pivot-shift test findings) were included if a proximal or midsubstance tear was confirmed by magnetic resonance imaging and if surgical treatment was possible within 3 weeks after the injury. Patients with previous knee injuries of the affected or contralateral knee or concomitant lesions such as meniscal tears, cartilage injuries, and collateral ligament injuries that would alter the operative procedure or the postoperative rehabilitation program were excluded. Stable meniscal lesions that neither required surgical treatment nor affected the rehabilitation protocol did not lead to exclusion. A block randomization protocol (n = 4 per block) was used to assign patients to undergo either ACL repair with DIS (Ligamys; Mathys Medical) or ACL reconstruction with an ipsilateral semitendinosus tendon autograft. A resident, who was not involved in the study, opened a sealed envelope just before surgery to allocate the patients to one of the treatment groups.

### Surgical Technique and Rehabilitation

Operative procedures were performed by 4 authors of the study who are all experienced in ACL surgery (M.J.R., M.H., C.K., B.S.). DIS was performed according to a technique described previously^28,29^: four 2.0 polyester sutures were passed through the ACL stump, and a K-wire was placed at the posterior edge of the tibial ACL footprint with a standard 60° tibial aiming device. The tibial monoblock was implanted after K-wire–guided drilling of the implant side. Care was taken to preserve a minimum of 2 cm of bone substance between the joint line and the implant. The sutures of the ACL stump were shuttled through a 2.3-mm drill hole at the femoral ACL insertion side. Then, a braided cord containing the femoral button was shuttled through the joint in a retrograde fashion and fixed to the tibial monoblock with a pretension of 80 N, close to full knee extension. Intraoperatively, we found 4 midsubstance ACL tears and 39 proximal ACL tears in the ACL repair group (Table 1). However, after repair, ACL stumps were closely adapted in each case.

**Table 1:** Patient Characteristics at Baseline and at 5-Year Follow-up^*a*^

	Baseline		5 y	
Characteristic	Repair (n = 43)	Reconstruction (n = 42)	Repair (n = 34)	Reconstruction (n = 30)
Sex, male/female, n	25/18	31/11	19/15	22/8
Age, y	28.7 ± 11.4	27.6 ± 10.6	28.3 ± 11.5	27.1 ± 11.5
Tegner score before injury	5.9 ± 1.5	6.6 ± 1.7	5.6 ± 1.2	5.9 ± 1.0
Smoker, yes/no, n	4/39	5/37	2/32	3/27
Body mass index, kg/m^2^	23.0 ± 2.0	24.6 ± 2.8	22.7 ± 2.1	23.8 ± 2.4
Time from trauma to surgery, d	14.5 ± 5.2	16.2 ± 7.3	14.1 ± 5.1	15.2 ± 6.4
Tear location, proximal/midsubstance, n	39/4	32/10	31/3	24/6

a Data are reported as mean ± SD unless otherwise specified.

Anatomic single-bundle ACL reconstruction was performed using a 4-strand semitendinosus tendon autograft with femoral fixed-loop suture button fixation and tibial hybrid fixation (interference screw and cortical suture button).^39^ Debridement or partial resection of small meniscal lesions was performed in 12 cases in the ACL repair group and 6 cases in the ACL reconstruction group. These lesions did not alter postoperative rehabilitation, and these patients were therefore not excluded. No intraoperative complications were encountered in either group. At the end of surgery, the affected knee was immobilized in a stiff brace at 0°.

A co-contraction routine of the quadriceps and hamstring muscles was practiced starting on day 1 after surgery. At 5 days after the intervention, a brace-free rehabilitation program was initiated including nonweightbearing exercises without limitations in range of motion. After 2 weeks of partial weightbearing (20 kg), full weightbearing was allowed, and quadriceps and hamstring strength training was started with closed-chain knee exercises. Starting at week 4, proprioceptive exercises including dynamic stability training were performed. Straight-line running was allowed after 6 weeks. Pivoting and competitive sports were allowed after at least 8 months if a return-to-sports test (ACL–Return to Sport after Injury scale, Knee injury and Osteoarthritis Outcome Score Sport and Recreation subscale, bilateral quadriceps force test in a leg press machine, single-leg hop for distance, and speedy hop test) was successfully completed. Removal of the tibial implant after DIS was not performed regularly.

### Follow-up Examinations

**Figure 1.**
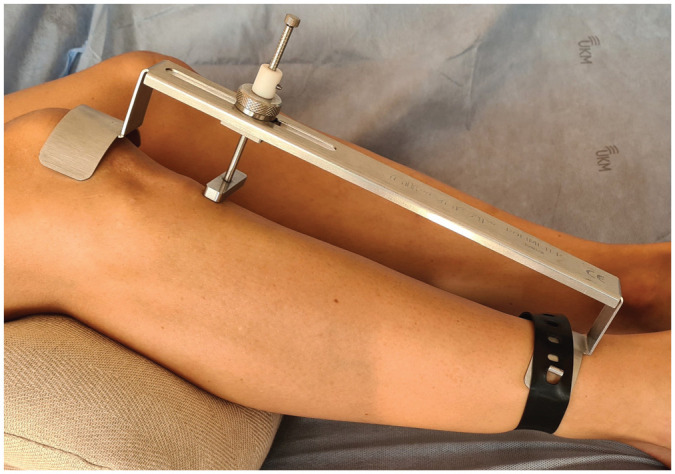
A Rolimeter (Aircast) was used to assess instrumented knee joint laxity with the knee in 30° of flexion.

Examinations were conducted at 6 weeks, 6 months, and 1, 2, and 5 years after index surgery by 2 examiners who were blinded and had not been involved in the operative treatment. Validated measurement tools were used to assess objective and subjective outcomes. Rolimeter (Aircast) testing with maximum manual pressure was performed with both knees in 30° of flexion to assess the side-to-side difference in ATT (ΔATT) at 1, 2, and 5 years postoperatively (Figure 1). The Tegner activity scale, the International Knee Documentation Committee (IKDC) subjective score, and the Lysholm score were administered at every follow-up examination. Recurrent instability was defined as ΔATT >3 mm or a positive pivot-shift test finding, each in combination with the subjective feeling of instability (“giving-way”). Patients with recurrent instability were excluded from further follow-up examinations. Adverse events and revision surgery during the follow-up period were recorded.

### Statistical Analysis

A power analysis before the study resulted in the necessity of enrolling 28 patients per group based on an expected mean ΔATT of 3 mm (regarded as clinically significant^31,35^), a standard deviation of 2 mm, and an estimated loss to follow-up of 15% (α = .05; β = 0.8). ΔATT measured with the Lachman/Rolimeter test was chosen for the power analysis because of its high sensitivity and diagnostic accuracy.^31,35^

Differences in scores between the different follow-up examinations within 1 group were analyzed with the Friedman test, and the Mann-Whitney *U* test was used to evaluate differences between the 2 intervention groups. The Bonferroni correction was applied to adjust the level of significance for multiple testing with significance set at *P* < .05.

## Results

**Figure 2.**
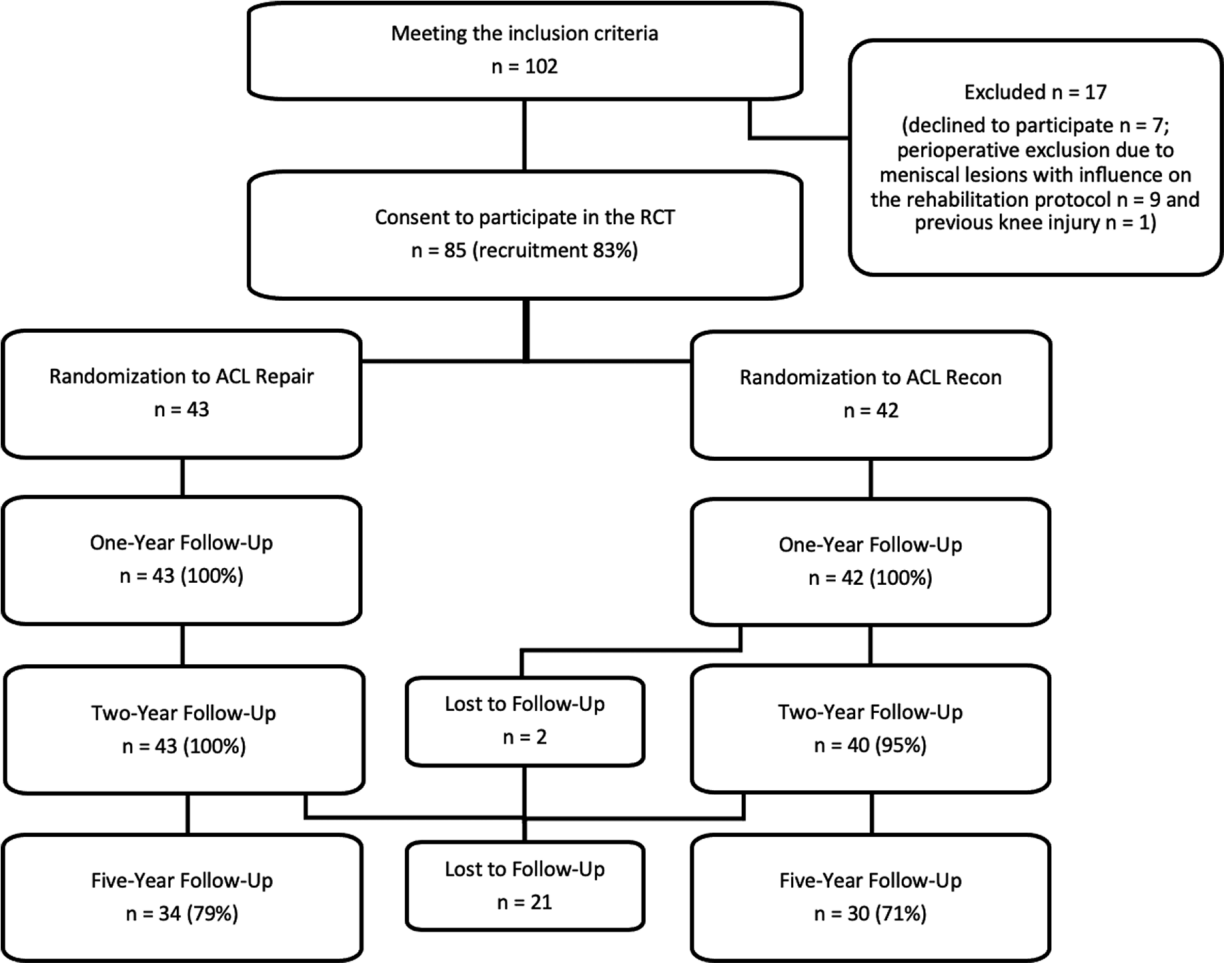
Diagram of grouping and patient flow. ACL, anterior cruciate ligament; RCT, randomized controlled trial; Recon, reconstruction.

A total of 102 patients with an acute isolated ACL tear and a scheduled operative procedure within 3 weeks after the injury were screened for eligibility. Overall, 85 patients gave their consent to participate in the study, resulting in a recruitment rate of 83%. As illustrated in Figure 2, after 5 years, 34 of 43 patients (79%) in the repair group and 30 of 42 patients (71%) in the reconstruction group were eligible for follow-up, with a follow-up rate of 75% (64/85). There were 21 patients (25%) who were not available for follow-up at 5 years for the following reasons: moved to a different country (n = 2) or region (n = 5) or did not respond to the follow-up invitations (n = 14).

The characteristics of both groups were comparable. Excluding those cases that were ineligible for follow-up at 5 years, the mean age was 28.3 ± 11.5 and 27.1 ± 11.5 years, respectively, and the mean Tegner score before the injury was 5.6 ± 1.2 and 5.9 ± 1.0, respectively, for the repair and reconstruction groups. The characteristics at baseline and the 5-year follow-up are summarized in Table 1.

### Instrumented Knee Joint Laxity

**Figure 3.**
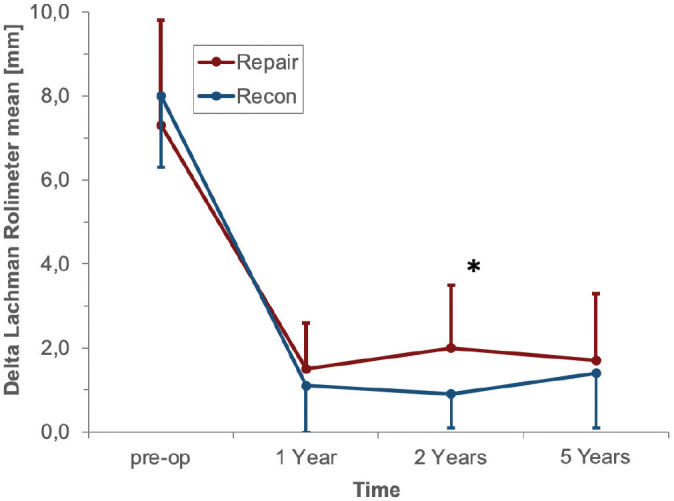
Anterior tibial translation presented as the difference between the injured and contralateral knees. *Significant difference between both groups (*P* = .009). Recon, reconstruction.

Before the intervention, ΔATT was 7.6 ± 2.6 mm in the repair group and 8.2 ± 1.4 mm in the reconstruction group. At 1 year after surgery, ΔATT was <3 mm in both groups, representing the successful treatment of anterior knee instability. At the 2-year follow-up, a significant difference in favor of the reconstruction group was found (*P* = .009), although ΔATT remained <3 mm in both groups (Figure 3). At 5 years, ΔATT was 1.7 ± 1.6 mm in the repair group and 1.4 ± 1.3 mm in the reconstruction group (*P* = .334).

### Patient-Reported Outcomes

**Figure 4.**
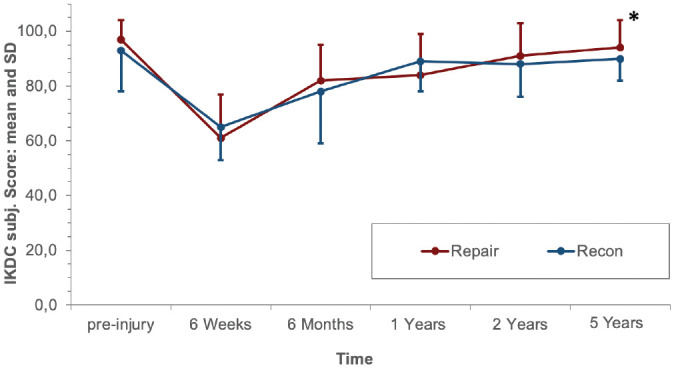
International Knee Documentation Committee (IKDC) subjective score of the 2 groups at the follow-up examinations. *Significant difference between both groups (*P* = .047). Recon, reconstruction.

**Figure 5.**
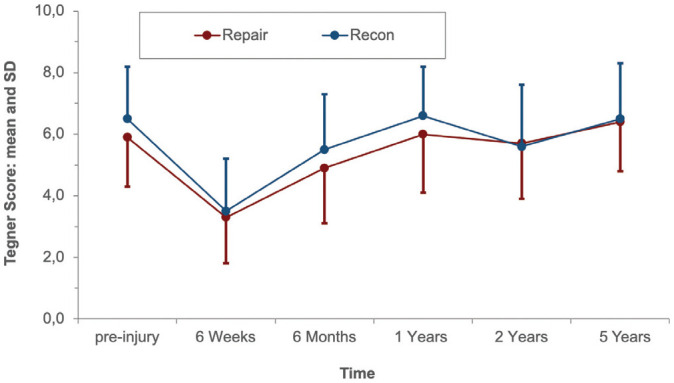
Tegner score of the 2 groups at the follow-up examinations. Recon, reconstruction.

**Figure 6.**
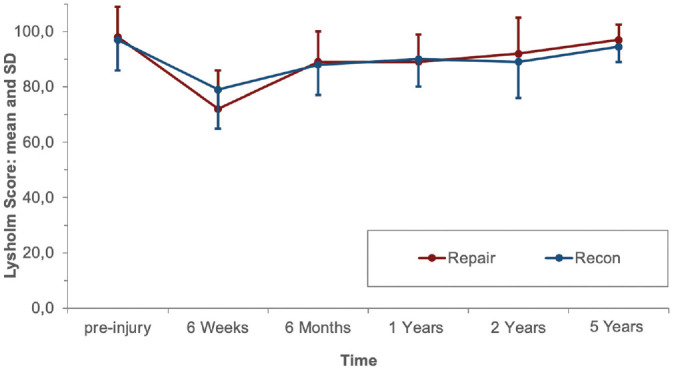
Lysholm score of the 2 groups at the follow-up examinations. Recon, reconstruction.

Starting from 6 weeks after surgery, PROs improved continuously until 1 year postoperatively and were maintained at the preinjury level at 2 and 5 years. At the 5-year follow-up, the mean Lysholm score was 97.0 ± 5.4 versus 94.5 ± 5.5 (*P* = .322), respectively, in the ACL repair group versus ACL reconstruction group. The mean IKDC subjective score was 94.1 ± 9.9 in the repair group and 89.9 ± 7.8 in the reconstruction group (*P* = .047). No statistically significant difference was found between the repair and reconstruction groups regarding the Tegner score (*P* = .423) at the 5-year follow-up. The PROs are shown in Figures 4 to 6.

### Recurrent ACL Instability and Revision Surgery

Recurrent instability was found in 12 of 34 patients (35%) after ACL repair and in 6 of 30 patients (20%) after ACL reconstruction at a mean time of 27 months (range, 7-57 months) and 15 months (range, 9-26 months), respectively. However, the difference between both groups was not statistically significant (*P* = .09). Overall, 10 of 12 patients with recurrent instability in the ACL repair group had a preinjury Tegner score ≥7 and were younger than 25 years at the time of the injury. In the ACL reconstruction group, all 6 patients with recurrent instability were younger than 25 years, and 5 of 6 had a preoperative Tegner score ≥7 (Table 2). In the ACL reconstruction group, 1 patient had ΔATT of 4 mm at 2 and 5 years; in the absence of subjective instability, the patient was classified as a coper (preoperative and postoperative Tegner score of 6) and was therefore included in the follow-up examinations. A contralateral ACL tear occurred in 2 patients in the ACL reconstruction group, whereas no contralateral ACL injury was found in the ACL repair group.

**Table 2:** Recurrent Instability at 5-Year Follow-up^*a*^

	No Recurrent Instability		Recurrent Instability	
	Repair (n = 22)	Reconstruction (n = 24)	Repair (n = 12)	Reconstruction (n = 6)
Age <25/≥25 y	4/18	5/19	10/2	6/0
Tegner score <7/≥7	18/4	16/8	2/10	1/5
Tear location, proximal/midsubstance	21/1	20/4	10/2	4/2

a Data are reported as No.

A total of 14 subsequent surgical procedures were performed in 13 patients (38%) in the ACL repair group. ACL revision surgery because of the failure of ACL repair was performed in 10 patients (29%) and consisted of a single-stage intervention with removal of the tibial monoblock and ACL reconstruction with an autologous hamstring tendon graft in each case. In 3 cases, additional suturing of a secondary meniscal lesion was performed. In the ACL reconstruction group, revision surgery was necessary in 8 patients (27%). ACL revision surgery was performed in 5 cases (17%) and consisted of staged revision because of tibial tunnel widening >10 mm in every case. During ACL revision surgery, repair of a secondary meniscal lesion was performed in 2 cases and autologous chondrocyte implantation in 1 case. Subsequent surgical procedures that were performed not because of recurrent ACL instability consisted of implant removal because of symptoms over the implant site (n = 3), arthroscopic surgery for secondary meniscal lesions (n = 3), and arthroscopic arthrolysis because of extension deficits (n = 2), with 4 cases in each study group. The differences between groups regarding revision surgery, revision ACL reconstruction, and non–ACL revision surgery were not statistically significant (Table 3).

**Table 3:** Subsequent Surgical Procedures at 5-Year Follow-up^*a*^

	Repair (n = 34)	Reconstruction (n = 30)	*P* Value
Patients with revision surgery^*b*^	13 (38)	8 (27)	.2483
Revision ACL reconstruction	10 (29)	5 (17)	.1182
Additional meniscal repair	3	2	
Additional cartilage repair	0	1	
Non-ACL revision surgery	4 (12)	4 (13)	.4264
Arthroscopic arthrolysis	1	1	
Secondary meniscal surgery	1	2	
Isolated implant removal	2	1	

a Data are reported as No. of patients or n (%). ACL, anterior cruciate ligament.

b In each group, 1 patient underwent non–ACL revision surgery before recurrent ACL instability occurred and was treated by ACL revision surgery.

## Discussion

In the present RCT, instrumented knee joint laxity after ACL repair with DIS was not inferior to that after ACL reconstruction, with ΔATT <3 mm in both groups at 5 years postoperatively. There were favorable PROs as soon as 1 year after surgery that plateaued until 5 years postoperatively in both groups, without the inferiority of ACL repair. Although no significant difference was found between repair and reconstruction, a critical appraisal of the rates of recurrent instability (35% vs 20%, respectively) and revision surgery (38% vs 27%, respectively) is needed.

Overall, 3 prospective controlled level 1 studies with follow-up periods until 2 years postoperatively have been published comparing ACL repair with DIS to ACL reconstruction.^22,28,43^ However, the midterm and long-term results of ACL repair with DIS have been evaluated in noncontrolled trials only.^1,2^ Therefore, the present study is the first to report the 5-year follow-up of ACL repair with DIS in a prospective randomized study.

In the present study, a significant difference regarding instrumented knee joint laxity at 2 years was found in favor of ACL reconstruction in comparison with ACL repair (ΔATT: 0.9 vs 1.9 mm, respectively; *P* = .009).^28^ However, no significant difference was found at 5 years between reconstruction and repair (ΔATT: 1.4 and 1.7 mm, respectively [*P* = .334]), and ΔATT was <3 mm in both groups at each follow-up, indicating no anterior knee instability in both groups.^3,31,35^ Accordingly, previous cohort studies had determined ΔATT to be between 1.4 and 2.1 mm at 2 and 5 years after ACL repair with DIS measured by the Rolimeter or KT-1000 arthrometer.^1,2^ However, the fact that both study groups were reduced by the exclusion of patients with treatment failure between the 1- and 2-year follow-up and the 2- and 5-year follow-up, respectively, should be considered when comparing ΔATT and PROs in each group over time in the present study (Figures 3-6).

Knee joint function assessed by PRO measures was reestablished after ACL repair with DIS as soon as 1 year postoperatively in prospective cohort studies, maintaining favorable scores until the 5-year follow-up.^1,2,37^ These findings are confirmed by the results of the present study, as no inferiority was found after ACL repair with DIS compared with ACL reconstruction regarding PROs during the entire follow-up period. At 5 years, a statistically significant difference in favor of ACL repair with DIS was found regarding the IKDC subjective score. However, the difference was below the minimal clinically important difference threshold and was therefore estimated to be not clinically relevant.^23^

Overall, 35% of the patients treated with ACL repair and followed until 5 years had recurrent ACL instability in the present RCT. Correspondingly, Ahmad et al^2^ reported a 70% five-year survival rate of ACL repair with DIS in a cohort of 57 patients. In the present study, the rate of recurrent instability increased from 2 to 5 years postoperatively from 16% to 35%, which is in accordance with cohort studies in which the rate of recurrent instability had been determined to be between 7.9% and 16% until 2 years postoperatively.^1,2,22,28,38^

Regarding ACL reconstruction, Andernord et al^4^ reported a recurrence rate of 1.8% at 2 years in a cohort of 16,930 patients from the Swedish national knee ligament registry. Fältström et al^11^ reported on recurrent instability in 4.3% of patients aged 27 years and 17.2% in younger patients (<16 years) within 5 years after the index procedure, indicating an increase in the recurrence rate with longer follow-up periods. However, rerupture rates up to 27% were found after ACL reconstruction in younger athletes and with longer follow-up periods.^4,11,12,26,44^ In the present study, the rate of recurrent instability at 5 years after ACL reconstruction with a hamstring tendon autograft was 20% and was not significantly more favorable in comparison with ACL repair with DIS. Although the rate of recurrent instability after ACL reconstruction in the present study was higher than in reports of graft failure after ACL reconstruction with a hamstring tendon graft in comparable age groups (up to 8%^40,42,45^), it was within the range of graft failure rates (up to 28.3%) in a recent systematic review of comparative studies by Belk et al,^5^ who revealed inconsistency in clinical outcomes at midterm follow-up after ACL reconstruction with hamstring tendon autografts.

Recurrent ACL instability was associated with younger age and increased preinjury activity levels in both groups of the present RCT (Table 2): 16 of 18 patients with recurrent instability were younger than 25 years, and 15 of 18 had a preinjury Tegner score ≥7. In recent studies, young age and a high preinjury Tegner score, along with midsubstance tears, were identified as the main risk factors for the failure of ACL repair.^18,30^ Furthermore, higher preinjury Tegner scores are associated with an earlier and higher level return to sports, which might further increase the risk of ACL reinjuries.^27^ However, these risk factors apply for both ACL repair and ACL reconstruction and therefore do not support reconstruction over repair in young and active patients.^4,18,46^

There were 2 cases of contralateral ACL tears that occurred after ACL reconstruction but no contralateral ACL tear after ACL repair. Considering that these numbers are too small to make any statement with confidence, they might indicate a potentially positive effect on proprioception by restoration of the native ACL. However, no corresponding superiority of ACL repair with DIS was shown regarding PROs.

In the present study, revision surgery was performed in 13 patients (38%) in the ACL repair group in comparison with 8 patients (27%) in the ACL reconstruction group. Revision rates of both study groups are within the range of published results.^4,17,48^ ACL revision surgery because of the failure of ACL repair was performed in 10 patients (29%). Single-stage revision with removal of the tibial monoblock and ACL reconstruction with autologous hamstring tendon grafts was performed in each case of failure of ACL repair. During ACL revision surgery, removal of the tibial implant of DIS left a cylindrical bone void of 10 × 23 mm. However, it has been shown that primary stability of tibial graft fixation in the case of failure of ACL repair with DIS is comparable with that of aperture fixation in primary ACL reconstruction if a bone stock of 20 mm is left between the DIS implant and the tibial joint line during the initial procedure.^16^

In contrast, each ACL revision surgery in the ACL reconstruction group consisted of staged revision because of tibial tunnel widening >10 mm according to our revision standard.^41^ However, when assuming a threshold of 15 mm, all the revision procedures would have been single-staged.

### Limitations

Several limitations need to be considered when interpreting the data of the current study. First, the follow-up rate was 75% at 5 years and therefore lower than expected. Because of the exclusion for recurrent instability (n = 18), only 46 of the 64 patients available for follow-up were included in the final assessment of instrumented laxity and PROs at 5 years. Therefore, at the 5-year follow-up, the study might be underpowered to determine differences between both groups regarding instrumented laxity, PROs, and treatment failure.

Second, the rate of recurrent instability was higher than expected in both groups, and although the difference was not statistically significant between ACL repair and ACL reconstruction, it is considered clinically relevant in both groups. The high rate of recurrent instability after ACL reconstruction is in accordance with the findings of inconsistent clinical outcomes at midterm follow-up with ACL reconstruction using hamstring tendon autografts by Belk et al.^5^ Quadriceps tendon or bone–patellar tendon–bone autografts are associated with lower failure rates and therefore increasingly used in young and active patients.^6^ Furthermore, anterolateral tenodesis has been proven to decrease the rate of recurrent instability when combined with ACL reconstruction.^15^ A similar effect may be assumed for combined ACL repair with additional anterolateral stabilization,^13^ although clinical data regarding this combined technique are pending.

Third, in the present study, patients with proximal and midsubstance tears were included. Recent studies have shown that ACL repair seems to be more successful with proximal-third tears.^18,30^ However, an influence of the tear pattern on the recurrence rate was not identified in this study (see Table 2).

Fourth, the operative procedures in both groups were performed by 4 different surgeons who are experienced with arthroscopic ACL reconstruction and were trained at the same center. Furthermore, all surgeons had performed the DIS procedure before the initiation of the study. No influence of the surgeon on recurrent instability and PROs was observed, in accordance with other studies.^19^

Fifth, the rehabilitation program of the present RCT with a 5-day period of immobilization, immediate range of motion training, and early full weightbearing may have influenced the risk of recurrent instability. However, the protocol was in accordance with current guidelines for rehabilitation after ACL repair,^34^ and brace-free rehabilitation and early weightbearing do not seem to have a negative effect on knee laxity and PROs.^33,34^

The concern of high rates of recurrent instability after ACL repair from previous studies^8^ has not been ruled out by the results of the present RCT. However, there is evidence that patient selection may significantly reduce the rate of recurrent instability after ACL repair with DIS.^17,18^ Henle et al^18^ found a recurrence rate of only 3.9% for proximal ACL tears in combination with a preinjury Tegner score <7, which is within the range reported for ACL reconstruction.^4,11,47^ Accordingly, and considering the previously mentioned limitations, the present study has shown that ACL repair with DIS may yield comparable knee joint stability and PROs in comparison with ACL reconstruction and is therefore considered a feasible option to treat acute ACL tears in patients aged ≥25 years with low to moderate activity levels (Tegner score <7).

## Conclusion

At 5 years after ACL repair with DIS, instrumented knee joint laxity and PROs were comparable with those after ACL reconstruction. The rate of recurrent instability was 35% in the ACL repair group in comparison with 20% in the ACL reconstruction group, with no statistically significant difference between both groups. Young age and a high preinjury activity level were the main risk factors for recurrent instability after both repair and reconstruction. Although ACL reconstruction remains the gold standard in the treatment of ACL tears, the present study supports the use of ACL repair with DIS as a feasible option to treat acute ACL tears in patients aged ≥25 years with low to moderate activity levels (Tegner score <7).
